# Design principles of the sparse coding network and the role of “sister cells” in the olfactory system of Drosophila

**DOI:** 10.3389/fncom.2013.00141

**Published:** 2013-10-23

**Authors:** Danke Zhang, Yuanqing Li, Si Wu, Malte J. Rasch

**Affiliations:** ^1^State Key Laboratory of Cognitive Neuroscience and Learning & IDG/McGovern Institute for Brain Research, Beijing Normal UniversityBeijing, China; ^2^School of Automation Science and Engineering, South China University of TechnologyGuangzhou, China; ^3^Center for Collaboration and Innovation in Brain and Learning Sciences, Beijing Normal UniversityBeijing, China

**Keywords:** drosophila olfactory system, homotypical projection neurons, noise robustness, mushroom body, divergent projection, sparse coding

## Abstract

Sensory systems face the challenge to represent sensory inputs in a way to allow easy readout of sensory information by higher brain areas. In the olfactory system of the fly *drosopohila melanogaster*, projection neurons (PNs) of the antennal lobe (AL) convert a dense activation of glomeruli into a sparse, high-dimensional firing pattern of Kenyon cells (KCs) in the mushroom body (MB). Here we investigate the design principles of the olfactory system of drosophila in regard to the capabilities to discriminate odor quality from the MB representation and its robustness to different types of noise. We focus on understanding the role of highly correlated homotypic projection neurons (“sister cells”) found in the glomeruli of flies. These cells are coupled by gap-junctions and receive almost identical sensory inputs, but target randomly different KCs in MB. We show that sister cells might play a crucial role in increasing the robustness of the MB odor representation to noise. Computationally, sister cells thus might help the system to improve the generalization capabilities in face of noise without impairing the discriminability of odor quality at the same time.

## 1. Introduction

Sparse coding is a common computational strategy in neural systems (Olshausen and Field, 2004; Barak et al., 2013). For instance, it was shown that maximizing sparseness results in the emergence of receptive fields in model simulations which are strikingly similar in structure to that found in the visual system of primates (Olshausen, 1996; Olshausen and Field, 1997). Moreover, sparse codes were suggested to be important for memory [in the hippocampal CA3 region (Thompson and Best, 1989)], the auditory system (DeWeese et al., 2003), or the vocal tract of songbirds (Hahnloser et al., 2002). In insects, olfactory representations in the mushroom body (MB) by Kenyon cells (KCs) are also sparse (Perez-Orive et al., 2002; Heisenberg, 2003; Huerta et al., 2004; Jortner et al., 2007; Wessnitzer et al., 2007; Turner et al., 2008).

Sparse codes help to separate or decorrelate similar sensory input patterns, so that the discrimination of distinct sensory inputs becomes easier for a subsequent neural system processing sensory information. However, the capacity becomes limited in very sparse representation. For instance, in an ultimate sparse code, where a binary activation of a single neuron represents a distinct sensory input, at most *N* representations can be distinguished. In consequence, very sparse codes become sensitive to noise, because a random activation is easily misinterpreted as another odor quality. Thus, the ability to generalize to noisy sensory inputs can be poor for very sparse codes.

To overcome some of these limitations, sparse codes in neural systems are typically combined with divergent projections from lower sensory areas to higher areas to increase capacity through the number of neurons. For instance, in the human visual system, the ratio of LGN to V1 cells is 1:40 (Wandell, 1995). Similarly, the ratio of the number of glomeruli in the antennal lobe (AL) to the number of KCs in the mushroom body (MB) is also 1:40 (Hallem and Carlson, 2006). However, while increasing the number of neurons increases the capacity of sparse representations, additional mechanism might have to be implemented to improve the generalization capabilities of the network to noisy sensory inputs.

One way to investigate these constraints in a model network is to investigate whether the MB activation patterns change in response to noise and compute a measure whether two patterns can generally be discriminated with a given sparsity and connection structure. A previous study examined the constraints of the connection structure on discriminability in the locust olfactory system (García-Sanchez and Huerta, 2003). The authors proposed an interesting mathematical framework and found a number of constraints on the design of the AL to MB projection in locusts. We here adapt and expand this framework to the particularities of the olfactory system of the fly *drosophila melanogaster* to derive its constraints on good discriminability of odor qualities and further use the network model to investigate the robustness of the olfactory system to noise.

In flies, when stimulated with a particular odor, a number of different types of olfactory receptor neurons (ORN) are activated (Hallem and Carlson, 2006). Because axons from ORNs converge into just one dedicated glomerulus in AL for each receptor type (Wilson and Mainen, 2006), a given odor will activate a certain number of glomeruli. Although there exist mechanisms for decorrelation of the ORN activity patterns to different odors, such as lateral processing and divisive normalization (Olsen and Wilson, 2008; Olsen et al., 2010), an odor representation in the antennal lobe can still be regarded as relatively dense. Typically, a rather large proportion of neurons fire when processing a single odor quality. It was found that 30–50% glomeruli are activated per odor (Hallem and Carlson, 2006). In contrast, when projection neurons (PN) project sensory inputs further from the glomeruli to the KCs of the mushroom body (Wilson and Mainen, 2006), only a small percentage of the ca. 2000 (Turner et al., 2008; Aso et al., 2009) KCs are active, resulting in a sparse representation of odor quality in MB.

While the general architecture of the olfactory system of the fly is similar to that of the locust, there also exist important differences. First, in flies the number of glomeruli is relatively small [50 compared to e.g., 830 in locust (Leitch and Laurent, 1996; Chou et al., 2010)]. More importantly, in contrast to the locust, each glomerulus in flies contains on average about 3 [2–5 (Stocker et al., 1990)] homotypical PNs that show almost identical activity pattern caused by shared input and gap junction coupling (Kazama and Wilson, 2009; Huang et al., 2010; Yaksi and Wilson, 2010). Because of their high correlation, we here call these PNs “sister cells.” Despite almost identical firing behavior, these homotypic PNs project randomly to different target KCs in the mushroom body (Masuda-Nakagawa et al., 2005; Kazama and Wilson, 2009). Similar types of sister cells have been characterized in other species such as frog (Chen et al., 2009) and mice (Dhawale et al., 2010; Padmanabhan and Urban, 2010; Tan et al., 2010). However, their function in general, or their usefulness to each individual species in particular, remains unclear.

We here put forward the hypothesis that sister cells in flies help the olfactory system to increase the robustness to noise and therefore help to stabilize the odor representation of the KCs in MB. We show that strong gap junction coupling between sister cells is crucial for their influence on the robustness of the system. Depending on the assumed noise strength and tolerance thresholds of the system, we found that already a few sister cells per glomeruli increase the system's robustness to noise considerably.

By calculating the probability of the expected similarity of representations of distinct odors in MB, we further derived analytical equations for the discrimination capabilities without the explicit use of classifiers or readouts, and give constraints on the connectivity for a given sparseness level in MB.

## 2. Results

The Result section is structured as follows. After introducing our network model of the olfactory system of drosophila, we first investigate the robustness of the MB odor representation to noise and highlight the role of sister cells. Then we analyze constraints on network parameters resulting from requiring a good discrimination ability of odors in MB.

### 2.1. Network model

During stimulation by an odor, a number of different types of olfactory receptor neurons (ORN) are activated. Because axons from ORNs of particular type converge into just one dedicated glomerulus in the antennal lobe (Wilson and Mainen, 2006), a given odor will activate a certain number of glomeruli. In our mathematical derivation, we assume that activity levels of glomeruli are binary, they are either activated by an odor or not. In simulations of Section 4.3, we consider graded neural responses.

In each of the *N*_*G*_ glomeruli, *M* highly correlated PNs (sister cells) receive the ORN's input and project to several of the *N*_*K*_ KCs in the mushroom body. We assume that all sister cells of a glomerulus behave identically to an odor, but may project to different KCs. The projection targets KCs randomly (Masuda-Nakagawa et al., 2005; Kazama and Wilson, 2009; Caron et al., 2013) in an independent fashion with a connection probability *p*_*c*_. Thus the probability of having *C* synaptic connections to a KC is given by the binomial distribution

(1) $$p(C)=\left(\begin{array}{c} MN_{G}\\ C \end{array}\right)p_{c}^{C}{\left(1-p_{c}\right)}^{MN_{G}-C}$$

Therefore, each KC receives on average 〈*C*〉 = *p*_*c*_*N*_*G*_*M* synaptic connections from PNs.

Due to divisive normalization by the total amount of ORN input, it was found that the total activity of all PNs to a given odor is approximately constant (Olsen and Wilson, 2008; Olsen et al., 2010; Luo et al., 2010). In the case of binary activation levels, constant total PN activity amounts to a constant number of activated glomeruli for a given odor. We thus assume that each odor activates exactly *A* glomeruli.

Following an early study investigating the olfactory system of locusts (García-Sanchez and Huerta, 2003; Huerta et al., 2004), we emphasize the analysis of the anatomical structure of the olfactory system and therefore simplify dynamical aspects. All PNs project their activation state to their targeted KCs. If the number of synaptic inputs reaches a firing threshold θ, a KC will fire. For simplicity, we assume that a KC has only one of two states: quiescent (0) or firing (1).

With these assumptions, we can write the response *y*_*i*_ of the *i*th KC in the following form [McCulloch-Pitts neuron (McCulloch and Pitts, 1943)]:

(2) $$y_{i}=\{\begin{array}{ll} 1, & \sum_{j}w_{ij}x_{j}\geq \theta \\ 0, & \text{otherwise} \end{array}$$

where *x*_*j*_ ∈ {0, 1} denotes the *j*th PN's response state to an odorant stimulus. In our framework, connections do either exist, *w*_*ij*_ = 1, or not, *w*_*ij*_ = 0. If the number of connections received by a KC is *C*, it is ∑_*j*_ *w*_*ij*_ = *C*.

**Figure 1:**
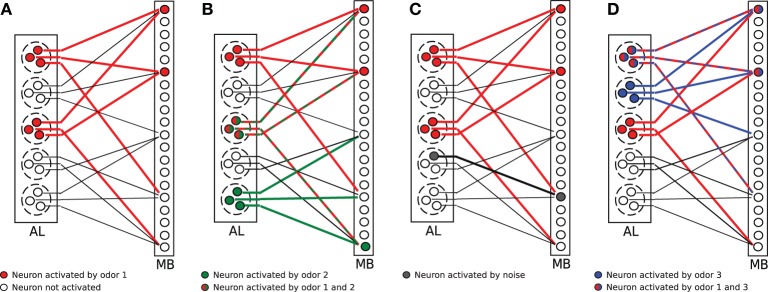
**Illustration of the olfactory network structure. (A)** An odorant pattern is represented by *A* randomly activated glomeruli in AL (red circles indicate activated PNs). All sister cells in activated glomeruli project to random KCs in the mushroom body (MB). The firing threshold of KCs is set to θ = 2 in this example. Two KCs get activated (red circles). **(B)** Two odors (red and green activations) overlap in the glomeruli activation, but the representation in MB might still be distinct. **(C)** Extrinsic noise is modeled as random activation of silent PNs (gray circle in AL). This synaptic input noise might induce a change in the activity pattern of KCs (gray circle in MB). **(D)** Faithful information transmission. Two odors (blue and red) might differ in glomeruli activation, but this information is lost in MB because the same selection of KCs are activated by chance.

The model network is illustrated in Figure 1A. In the cartoon, *A* = 2 glomeruli (dashed circles) of the antennal lobe (AL) got activated by an odor. Thus, all sister cells (here *M* = 3) of the activated glomeruli changed into the firing state (red circles). All sister cells project randomly into the mushroom body (MB). For illustration purposes, each KC receives exactly *C* = 3 connections here (solid lines; connections are only plotted for every fifth KC). The firing threshold in Figure 1 is arbitrarily set to θ = 2, so that those KCs which receive two or more activated connections (red lines) activate in turn, resulting in an corresponding odor representation in MB (red circles in MB).

### 2.2. Firing probability of kenyon cells

We start by deriving the KC firing probability from combinatorial arguments. Since all incoming connections are from random PNs, the probability of the amount of synaptic inputs received by any KC is identically distributed and uncorrelated to others.

According to our assumptions, there are exactly *MA* active PNs in the AL a KC could receive input from. For a KC cell to receive exactly *n* active presynaptic inputs, the KC should have a connection from exactly *n* of the *MA* cells. Because there are $\left(\begin{array}{c} MA\\ n \end{array}\right)$ combinations of connections to receive *n* connections from *MA* cells and the probability for a connection is *p*_*c*_ = 〈*C*〉/(*N*_*G*_*M*), we can write the synaptic input distribution *p*(*n*) as the binomial distribution

(3) $$p(n)=\left(\begin{array}{c} MA\\ n \end{array}\right)p_{c}^{n}{\left(1-p_{c}\right)}^{MA-n}$$

In other words, *p*(*n*) is the probability of a KC having exactly *n* activated presynaptic neurons during presentation of an arbitrary odor. Since each activated presynaptic neuron delivers an input of size 1 to the postsynaptic neuron and inputs are simply added in our neuron model (see Eq. 2), *p*(*n*) describes the probability of a KC to have exactly *n* synaptic inputs in response to an odor.

**Figure 2:**
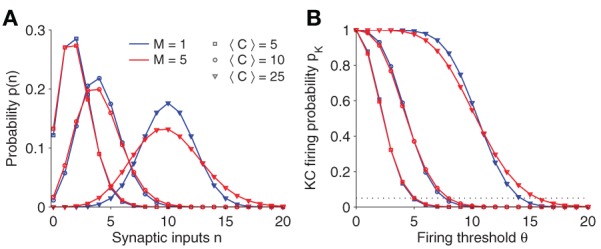
**(A)** Synaptic input distribution *p*(*n*). The distribution shifts and gets broader for increasing connectivity (〈*C*〉). Note that in case of multiple sister cells per glomerulus (red lines; *M* = 5) and fixed connectivity 〈*C*〉, its variance increases as well. The binomial distribution approximates a Gaussian for larger 〈*C*〉. **(B)** KC firing probability *p*_*K*_ vs. firing threshold θ. Note that the slope changes for different parameter. The threshold θ is usually set so that the KC firing probability is fixed at 0.05 (dotted line). Parameter: *N*_*G*_ = 50, *A* = 20.

Figure 2A plots the synaptic input distribution *p*(*n*) for a number of parameter settings. Naturally, if the average number of connections 〈*C*〉 is increased, the mean synaptic input increases as well. Note, however, that even if 〈*C*〉 is fixed, the shape of the distribution broadens slightly for increasing number of sister cells *M*.

In response to synaptic inputs, a KC will activate if the amount of input exceeds a threshold θ. Therefore, the KC firing probability *p*_*K*_ is given as

(4) $$p_{K}(\theta )=\sum_{n\geq \theta }p(n).$$

See Figure 2B for a plot of *p*_*K*_(θ). Generally, the firing threshold should be set large enough to ensure a low firing probability and a sparse activation pattern in KCs (dashed line in Figure 2B corresponds to *p*_*K*_ = 0.05).

### 2.3. Robustness to intrinsic noise

Noise is ubiquitous in neural systems. If the firing probability is very low in the MB to achieve a sparse representation of odors, a noise induced change of the firing state of even a few neurons might have a large impact. We wondered how the odor representation of KCs is affected by noise and whether the sister cells could promote robustness to some forms of noise.

In the following two sections, we consider two types of noise, intrinsic and extrinsic noise, respectively. Intrinsic noise refers to an accidental perturbation of an internal parameter, for instance a change in the firing mechanism. Extrinsic noise refers to fluctuations of the synaptic inputs. To be able to compare noise effects for different parameter values we will assume that the KC firing probability is given and maintained while changing parameters.

Let us first consider intrinsic noise. We model intrinsic noise by perturbing the firing threshold of a KC neuron. If the firing threshold is reduced by Δθ, a KC will fire more likely. The noise induced change of the firing probability Δ*p*^intr−^_*K*_ is given by summing up the probabilities of the additional synaptic inputs *n* = θ − Δθ,…,θ − 1 which result in a spike after perturbation:

(5) $$\Delta p_{K}^{\text{intr}-}=p_{K}(\theta -\Delta \theta )-p_{K}(\theta )=\sum_{n=\theta -\Delta \theta }^{\theta -1}p(n).$$

Analogously, if the threshold is increased by an amount Δθ, the firing probability of KCs is lowered and can be calculated as

(6) $$\Delta p_{K}^{\text{intr}+}=p_{K}(\theta +\Delta \theta )-p_{K}(\theta )=-\sum_{n=\theta }^{\theta +\Delta \theta -1}p(n).$$

To get a better intuition of the involvement of parameters in the intrinsic noise, we approximate the binomial distribution of *p*(*n*) by a Gaussian distribution *N*(μ,σ) with same mean and variance (which is a good approximation for larger 〈*C*〉, see Figure 2A). For Eq. 5 one finds (Eq. 6 can be approximated in analogous manner):

(7) $$\;\mu =MAp_{c}$$

(8) $$\sigma^{2}=MAp_{c}(1-p_{c})$$

with synaptic connection probability *p*_*c*_ = 〈*C*〉/(*MN*_*G*_). When the sum in Eq. 5 is approximated by an integral, we find

(9) $$\Delta p_{K}^{\text{intr}-}\approx \int_{\theta -\Delta \theta }^{\theta }\frac{1}{\sqrt{2\pi }\sigma }e^{-\frac{{\left(x-\mu \right)}^{2}}{2\sigma^{2}}}dx$$

(10) $$\;=\int_{\alpha -\frac{\Delta \theta }{\sigma }}^{\alpha }\frac{1}{\sqrt{2\pi }}e^{-\frac{x^{2}}{2}}dx.$$

where the firing threshold in Eq. 10 was set in terms of the mean and the variance of the (assumed Gaussian) synaptic input distribution θ = μ + α σ with α > 0 to ensure constant mean activity of the KCs. If Δθ is small, we can approximate the integral in Eq. 10 and find

(11) $$\Delta p_{K}^{\text{intr}-}\approx \frac{\Delta \theta }{\sigma }\frac{e^{-\frac{\alpha^{2}}{2}}}{\sqrt{2\pi }}$$

(12) $$\;=\frac{\Delta \theta }{\sqrt{MAp_{c}(1-p_{c})}}\gamma$$

(13) $$\;=\frac{\Delta \theta }{\sqrt{Ac(1-c/M)}}\gamma$$

by using the definition Eq. 8, abbreviating a term related to the mean activity, $\gamma \equiv \exp(-\alpha^{2}/2)/\sqrt{2\pi }$, and setting *c* ≡ 〈*C*〉/*N*_*G*_.

**Figure 3:**
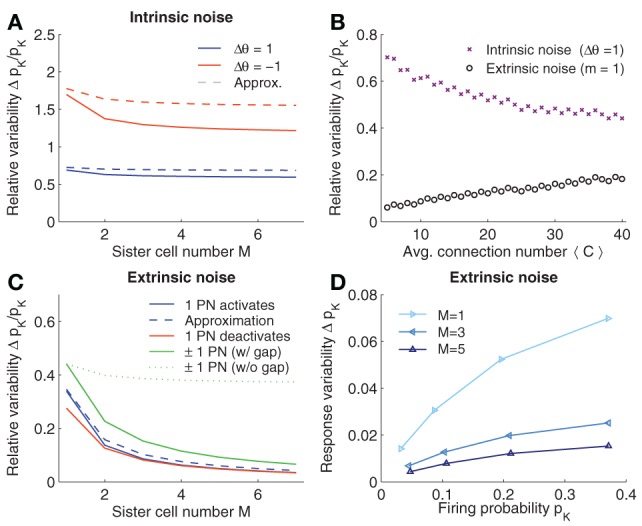
**Robustness to noise. (A)** Intrinsic noise modeled by varying the firing threshold of KCs by Δθ. Relative change in firing probability of KCs is plotted vs. the number of sister cells *M*. The Gaussian approximation Eq. 10 in dashed lines. **(B)** Relative noise induced variability with average number of connections 〈*C*〉. Varying 〈*C*〉 has opposite effects for extrinsic (*m* = 1 see panel **C**) and intrinsic noise. **(C)** Extrinsic noise. The effect of activating a single quiescent PN (blue curve) or deactivating an active PN (red curve) on the firing probability of KCs. Dashed curve shows the Gaussian approximation Eq. 20. General extrinsic noise where some PNs activate and others deactivates simultaneously (green curves). If gap junctions are included (that is the noise strength remains constant with *M*, i.e., *m*_1_ = 1 and *m*_2_ = 1) the induced KC variability reduced quickly (solid line), whereas if gap junctions are not considered (and thus the noise strength grows with *M*, i.e., *m*_1_ = *M*, *m*_2_ = *M*) the variability only slightly decreases (dotted line). **(D)** Variability of *p*_*K*_ induced by extrinsic noise (*m*_1_ = 1 and *m*_2_ = 1) depends on *p*_*K*_. Parameters (if not varied): *N*_*G*_ = 50, *A* = 20, 〈*C*〉 = 10, *M* = 3, and *p*_*K*_ ≈ 0.05.

Assuming *p*_*c*_ < 0.5 and a fixed KCs firing probability, Eq. 12 shows that the variability decreases if the connection probability *p*_*c*_ is increased. Similarly, increasing the number of connections reduces the variability (see Figure 3B; crosses).

Interestingly, a higher number of sister cells *M* per glomeruli reduces the intrinsic noise. When relating the case of *M* sister cells to without sister cells, the relative reduction of Δ*p*^intr−^_*K*_ is $\sqrt{\left(1-c)/(1-c/M\right)}$ (assuming constant 〈*C*〉; see Eq. 13). This reduction is moderate and saturates for large *M*. With *c* = 〈*C*〉/*N*_*G*_ = 10/50 = 1/5, the relative change in Δ*p*^intr−^_*K*_ becomes $2/\sqrt{5-1/M}$ which goes to $2/\sqrt{5}\approx 0.9$ for large *M*. Thus, while the amount of sister cells reduces the effect of intrinsic noise, the improvement relative to the case without sister cells does not exceed 10%.

In Figure 3A, the noise induced variability of the firing probability is plotted for varying *M*. For better comparison, we fixed the average number of synaptic inputs 〈*C*〉. Noise reduction with increasing numbers of *M* is slightly higher than the 10% estimated from the approximation (Eq. 12), however, the effect of sister cells on intrinsic noise remains small and saturates as predicted.

Note that we modeled intrinsic noise by changing the firing threshold in a single KC and looked at its change of firing probability. How frequent such threshold fluctuation occurs in individual cells of the large population of KCs in MB is not known. However, the qualitative behavior and the effect of parameters on robustness to noise should not change when regarding the whole population or only a single cell.

### 2.4. Robustness to extrinsic noise

Extrinsic noise in our model describes the situation when synaptic inputs to KCs have been perturbed: noise changes the activation level of some PNs. Since synaptic inputs might differ due to noise, KCs in turn might change their firing (see Figure 1C for an illustration).

To compute the effect of extrinsic noise, we randomly assign *m* quiescent PNs in the antennal lobe to be active. We will later analyze the case where both possibilities, activation of quiescent PNs and deactivation of active PNs, are present.

How would the activation of *m* quiescent PNs affect a KC in its firing? For that, one has to calculate the probability *p*(*l*|*m*) that *l* of the *m* randomly activated PNs project to a given KC. This problem is the same as for Eq. 3 and is given by a binomial distribution

(14) $$p\left(l|m\right)=\left(\begin{array}{c} m\\ l \end{array}\right)p_{c}^{l}{\left(1-p_{c}\right)}^{m-l}$$

Having *l* noise inputs to the KC will increase the total synaptic input of previously *n* by *l*. However, only if these additional noise inputs push the KC above threshold, i.e., *n* + *l* ≥ θ, will the firing probability be affected. Taking this into account, one finds

(15) $$\Delta p_{K}^{\text{extr}+}=\sum_{n=\theta -m}^{\theta -1}p(n)\sum_{l=\theta -n}^{m}p(l|m)$$

Analogous to the case of intrinsic noise, we approximate Eq. 15 to get a better intuition of the involvement of parameters. Using again a Gaussian distribution to approximate *p*(*n*), and set (analogous to above) θ = μ + ασ, one first finds

(16) $$p_{K}(\mu +\alpha \sigma )\approx \int_{\alpha }^{\infty }\frac{1}{\sqrt{2\pi }}e^{-\frac{x^{2}}{2}}dx.$$

Now suppose that *m* quiescent PNs are activated as a result of a noise perturbation. This will cause the synaptic input distribution to change slightly as the total amount of active PNs is increased, i.e., *MA* → *MA* + *m*. Hence,

(17) $$\;\mu^{\prime }=MAp_{c}+mp_{c},$$

(18) $${\sigma^{\prime }}^{2}=MAp_{c}(1-p_{c})+mp_{c}(1-p_{c}).$$

Computing the difference between the firing probability with and without noise yields the change of the firing probability caused by extrinsic noise. Using Eq. 16

(19) $$\Delta p_{K}^{{}^{\text{extr}+}}\approx \int_{\theta }^{\infty }\frac{1}{\sqrt{2\pi }\sigma^{\prime }}e^{-\frac{{\left(x-\mu^{\prime }\right)}^{2}}{2{\sigma^{\prime }}^{2}}}dx-\int_{\alpha }^{\infty }\frac{1}{\sqrt{2\pi }}e^{-\frac{x^{2}}{2}}dx$$

(20) $$\;=\int_{\beta }^{\alpha }\frac{1}{\sqrt{2\pi }}e^{-\frac{x^{2}}{2}}dx$$

with $\beta =\alpha \sqrt{\frac{MA}{MA+m}}-\frac{mp_{c}}{\sqrt{MA\;p_{c}(1-p_{c})+mp_{c}(1-p_{c})}}$. Since *m* is small, the integral can again be approximated resulting in

(21) $$\Delta p_{K}^{\text{extr}+}\approx (\alpha -\beta )\gamma$$

(22) $$\;\approx \left(\alpha -\sqrt{\frac{MA}{MA+m}}\alpha +\frac{mp_{c}}{\sqrt{MAp_{c}(1-p_{c})+mp_{c}(1-p_{c})}}\right)\gamma$$

(23) $$\;=\left(\alpha -\sqrt{\frac{MA}{MA+m}}\alpha +\frac{m}{\sqrt{\left(M/c-1)(MA+m\right)}}\right)\gamma$$

with the same *c* ≡ 〈*C*〉/*N*_*G*_ and γ as defined above.

Note first that the noise perturbation grows with the mean activity of the KCs (see also Figure 3D). From Eq. 22, we further find that, in contrast to intrinsic noise, reducing—instead of increasing—the average input connection 〈*C*〉 (or *p*_*c*_) results in a more robust noise response, if the firing activity in KCs is maintained (see Figure 3B).

Introducing sister cells, on the other hand, has again a positive effect on the robustness to noise. If we assume for the moment that *m* stays constant if *M* is changed (see Section 2.5), set 〈*C*〉 constant, and further assume *A* » *m* (so that *MA*/(*MA* + *m*) ≈ 1), we find that the relative change of Δ*p*^extr+^_*K*_ with *M* in respect to without sister cells is given by $\sqrt{\left(1-c)/(M(M-c)\right)}$, which is approximately 1/*M* as *c* is relatively small (*c* = 1/5). Thus, the improvement of the robustness to noise is considerable with increasing number of sister cells and indeed does not saturate for larger *M* in contrast to the case of intrinsic noise.

So far we have analyzed the case where *m* quiescent PNs activate in response to noise. In a similar manner we can derive the noise induced probability change when noise causes previously active PNs to turn off, and when both cases are combined (see Method section 4.2). The qualitative results are similar. The change in noise induced firing probability when activating or deactivating a single PN is plotted in Figure 3C for different values of sister cells *M*. Note that the approximation with a Gaussian, Eq. 20 (dashed line), is close to the exact values (solid lines). Note that the effect of sister cells on the robustness of the system to extrinsic noise is considerably, approximately halving the noise induced variations of the firing probability when *M* is doubled.

In conclusion, our results suggest that incorporating highly correlated sister cells into glomeruli enhances the robustness of the firing pattern in the mushroom body to both, extrinsic and intrinsic noise. On the other hand, connectivity structure has opposite effects on both noise types.

We further tested in simulations whether our theoretically derived results on the robustness to noise are applicable also when the assumption of binary activation in AL is relaxed to allow graded firing rate responses (see Method section 4.3). We found that the qualitative behavior of the reduction of noise with increasing number of sister cells as well as the effect of connectivity are very similar (compare to Figure 8).

### 2.5. Strong gap junction coupling between sister cells reduces the extrinsic noise

We found in the last section that the effects of extrinsic noise are reduced approximately by 1/*M* if sister cells are introduced.

However, we silently assumed that the number of PNs affected by the noise stays constant at *m*. However, if the number of sister cells is changed, the total number of PNs gets implicitly multiplied by *M* because additional PNs are introduced. In a larger pool of PNs, it is more likely to find a fixed number of *m* cells affected by noise; in fact *m* should be a certain fraction *f* of the total number of PNs, thus *m* = *fMN*_*G*_.

Thus *m* should be enlarged when adding sister cells. However, as described in the introduction, sister cells are highly correlated with each other by gap junction coupling. What would be the effect of noise on a system of strongly coupled sister cells?

To analyze this we built a simple rate model of *M* mutual connected sister cells and compared the variance of each cell's output rate to the variance of an injected independent Gaussian noise. As described in the following, we show that the variance of the noise gets reduced by a factor of 1/*M* for strong mutual coupling. Thus, gap junctions effectively reduce the risk of a sister cell to accidentally change its state.

We investigate the effect of the electrical gap-junction coupling of sister cells on extrinsic (added) noise. Within one glomerulus, we consider a group of *M* neurons receiving random external inputs with and without gap junctions between each other. In a stochastic differential equation formulation (Øksendal, 2003), the dynamics of an *i*th neuron's firing rate *r*_*i*_ can be written as

(24) $$\tau \frac{dr_{i}}{dt}=-r_{i}+f(I_{i})+w\sum_{j=1}^{M}\left(r_{j}-r_{i}\right)+\sigma \xi_{i}$$

where τ is the time constant of the neuronal firing rate. *I*_*i*_ is the input that is applied to the neuron, and *f* is the neuron's IO-function which concrete shape is not important for the discussion here. The strength of the gap-junction coupling between neurons is given by *w* and assumed equal for all neurons for simplicity. Extrinsic noise is modeled as a Gaussian process with zero mean and standard deviation σ. In Eq. 24, ξ_*i*_ is thus a Gaussian white noise variable with zero mean and unit variance.

In this rate model, we describe gap junctions as depending on the neuronal firing rate difference between neurons. This is a reasonable assumption because gap junctions are typically modeled as resistive connections dependent on the difference of somatic voltage between connected cells (e.g., Moortgat et al., 2000).

In the following, we compute the mean and the variance of the stochastic differential equation for the rate of the coupled neurons. Reorganizing the neural dynamics Eq. 24 into matrix form, we have

(25) τdr=−(Ur−b)dt+σdW

where *r* is a vector of length *M*, *W* is a *M*-dimensional Gaussian noise term (zero mean and unit variance matrix). The input term is *b*_*i*_ = *f*(*I*_*i*_) and the matrix *U* is defined as

(26) $$U=(1+Mw)I_{M}-w11^{T}$$

where **1** is the vector of only ones and *I*_*M*_ the identity matrix.

We note that Eq. 25 is a system of (inhomogeneous) linear stochastic differential equations (SDE) with independent Gaussian noise terms (having zero mean), symmetric *U*, and time-independent *U* and *b*. The evolution of the mean and variance of such a system of linear SDEs is well known (Øksendal, 2003; Jimenez, 2012) and the stationary solution for the mean and co-variance matrix of Eq. 25 is given by

(27) $$\begin{array}{l} \text{ E}(r)=b\\ \text{Var}(r)=\frac{\sigma^{2}}{2\tau }U^{-1}. \end{array}$$

Since the matrix *U* is a rank-1 update of a diagonal matrix, it turns out that its inverse can be written as

(28) $$U^{-1}=\frac{1}{1+Mw}I_{M}+\frac{w}{1+Mw}11^{T}.$$

Altogether, we thus find for the variance

(29) $$\text{Var}(r)=\frac{\sigma^{2}}{2\tau }\left(\frac{1}{1+Mw}I_{M}+\frac{w}{1+Mw}11^{T}\right)$$

**Figure 4:**
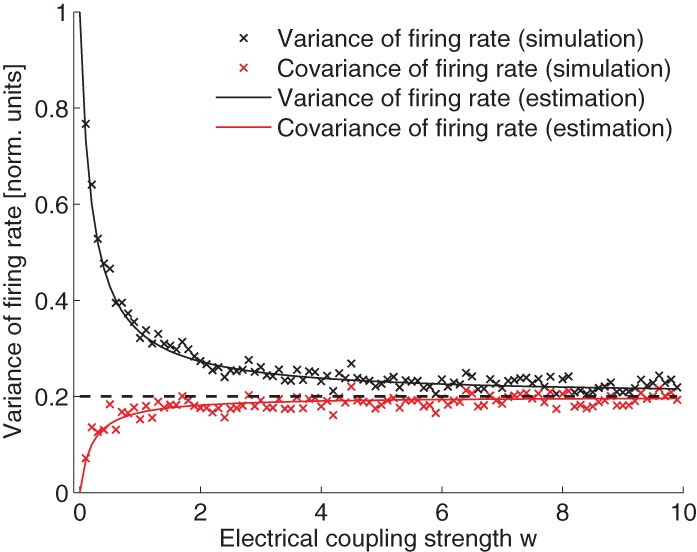
**Variance and covariance of the neuronal firing rate as a function of the electrical coupling strength.** Values are normalized by the expected variance without coupling, that is σ^2^/(2τ). Simulation and theoretical estimation show that the variance decays with coupling strength while covariance increases. In the limit of large coupling, variance and covariance are both equal to 1/*M* of the magnitude of the variance when there is no electrical coupling (dashed line). Parameters (since the variables can be scaled arbitrarily the units are omitted): *I* = 5; *M* = 5; σ = 0.2; τ = 10. IO-function *f*(*x*) = α *x* + β with α = 3, β = 5.

From Eq. 29, we can see that, when there is no electrical coupling, *w* = 0, the variance of the neuronal firing rate is σ^2^/(2τ) and the covariance of the neuronal firing rate is 0. When electrical coupling is extremely large, *w* → ∞, all variance and co-variance terms become equal and are given by σ^2^/(2*M*τ). Note that the variance is exactly reduced by 1/*M* as a result of electrical coupling. The variance and covariance terms are compared in Figure 4. The simulated values fit very well the theoretical calculations.

Taken together, the number of noise activated PNs *m* stays indeed constant if *M* is increased, because the gap-junction coupling reduces the likelihood of the noise induced changes by 1/*M*. In consequence, the number of accidentally activated PNs, *m*, is not dependent on *M* anymore. With strong gap junction coupling, *m* is thus a constant when varying *M*. In this sense, our calculation in the last paragraph indeed modeled extrinsic noise in a realistic setting.

Note that if one would increase the number of PNs *M*-fold (while keeping the fraction of activated glomeruli constant), but without having highly correlated sister cells in the system, the reduction of extrinsic noise would be much less effective since the probability of having a noise event would grow with the number of PNs, e.g., *m* ∝ *M*. This situation is plotted in Figure 3C in case of extrinsic noise with both silencing and activation events (dotted green line). On the other hand, including sister cells would mean *m* constant (regardless of *M*), so that the relative change in the firing probability of KCs decreases much more rapidly (Figure 3C; solid green line).

### 2.6. Sparse codes reduce overlaps in MB

In the previous sections, we examined the robustness to noise. From a computational perspective, robustness of the MB odor representation to noise improves the performance of a hypothetical readout of the sensory information. For instance, if a higher brain area has to decide whether two noisy patterns in MB result from an activation to the same odor, a more noise robust KC activation would make this generalization task easier.

Another mechanism to increase the generalization capabilities while maintaining discrimination performance at the same time is to require that odor representations do not share too many common activations for different odors. For instance, if two odor representations would be required to differ by the activation of at least three neurons, an incoming odor pattern with an accidental change in a single neuron would be still correctly classified (assuming that the margin of the classifier would be set accordingly). In contrast, if two odor representations differed by only one neuron, a pattern with an accidental change in a single neuron could instead be misclassified.

In this sense it should thus be beneficial to reduce the risk of large overlaps of odor representations when designing the olfactory system. In divergent projections, such as the AL to MB projection, it is well known that patterns in MB become generally less overlapping if activations are sparse. To investigate this property in our framework in a quantitative way, we compute the exact probability of finding shared neurons in two odor representations in MB for a given amount of shared activations in AL. Thus, we ask: for individual activation patterns, if we have a certain overlap in AL, what is the expected overlap in MB?

Assume that *X*_1_ = (*x*^1^_1_,…,*x*^*MN*_*G*_^_1_) and *X*_2_ = (*x*^1^_2_, …, *x*^*MN*_*G*_^_2_) are two {0, 1}-vectors with *MN*_*G*_ elements representing the PN activation patterns in response to two odors. Each odor activates exactly *A* glomeruli (thus *MA* PNs). However, some of the activated glomeruli might be identical (Wilson and Mainen, 2006) that is both odors potentially have an overlapping representation in AL. This situation is illustrated in Figure 1B (green and red circles). Let the number of commonly activated glomeruli be *X*_1_ · *X*_2_ = *Mo*. Since |*X*_1_| = |*X*_2_| = *MA* PNs are always activated, the overlap normalized by the expected number of activated PNs is

(30) $${\text{OV}}_{\text{AL}}(o)=\frac{o}{A}.$$

The PNs project to the KCs which in turn fire according to their synaptic input from the PNs. Let *Y*_1_ = (*y*^1^_1_,…,*y*^*N*_*K*_^_1_) and *Y*_2_ = (*y*^1^_2_,…,*y*^*N*_*K*_^_2_) be the activation patterns corresponding to *X*_1_ and *X*_2_ in MB. Note that *Y*_1_ and *Y*_2_ are {0, 1}-vectors with *N*_*K*_ elements (the number of KC cells). On average, according to the firing probability of KCs (*p*_*K*_; see Eq. 4), odors will activate *p*_*K*_*N*_*K*_ neurons in the mushroom body.

We asked how large the induced overlap of two odor representation in MB will be on average if the overlap between two odor representations in AL is known to be *o*.

Since the connectivity structure is independent for each KC, it is enough to consider a single KC. The probability of having a common activation of a single KC for both odors given an overlap *o* of their AL representations is *p*(*y*_1_ = *y*_2_ = 1|*o*). Thus, *N*_*K*_*p*(*y*_1_ = *y*_2_ = 1|*o*) is the expected number of shared KCs in the representation of the two odors in MB. Since *p*_*K*_*N*_*K*_ is the expected number of activated KCs in response to any odor, the (normalized) overlap in MB for a given AL overlap *o* is

(31) $${\text{OV}}_{\text{MB}}(o)=\frac{p(y_{1}=y_{2}=1|o)}{p_{K}}.$$

The probability *p*(*y*_1_ = *y*_2_ = 1|*o*) can indeed be exactly calculated by using a combinatorial approach. We give the derivations in the Method Section 4.1 (Eq. 39).

**Figure 5:**
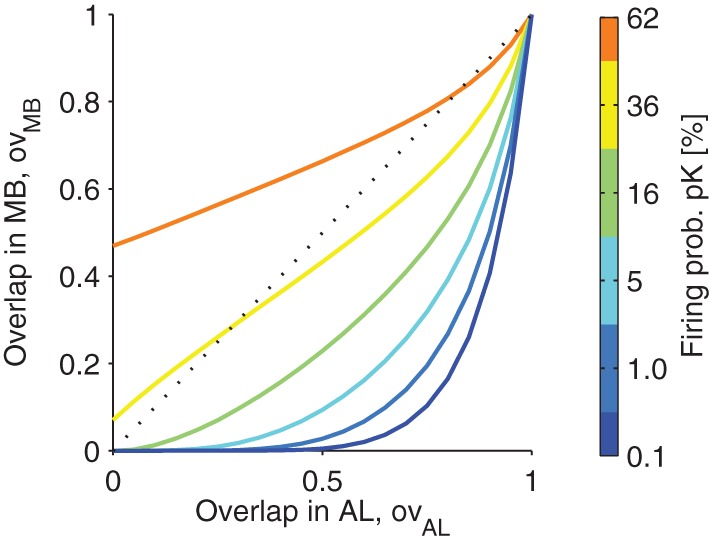
**The projection from antennal lobe to the mushroom body reduces the overlap of two odor representations for sparse codes.** Overlap in the mushroom body ov_MB_ is plotted as a function of the overlap of representations in the antennal lobe *ov*_AL_. Colors indicate different levels of KC firing probability *p*_*K*_ (related to the sparseness of the MB representations). Note that for low *p*_*K*_ even highly overlapping representations in the AL are very likely to be non-overlapping in MB. However, for higher firing probabilities *p*_*K*_, this effect diminishes. Parameters: *M* = 3, *N*_*G*_ = 50, *A* = 20, 〈*C*〉 = 10 (Note that with these parameters the firing probability in the AL is 40%).

In Figure 5, the relation of the overlap of the representation in MB is plotted as a function of the overlap in AL. Note that the function is highly sub-linear, showing that the overlaps of representations are reduced as long as the firing in MB is sparse (low firing probability *p*_*K*_; colors in Figure 5).

### 2.7. Probability of having distinct odor representations in MB

A challenge for the olfactory system is to ensure that sensory information can still be read out from the MB after projecting the AL odor representations to the MB. In particular, information about odor identity should not be lost. For instance, consider the situation illustrated in Figure 1D. Two odors, odor 1 (red), and odor 2 (blue), have different representations in AL, *X*_1_ and *X*_2_, with *X*_1_ ≠ *X*_2_. However, there is a chance that due to the particular structure of the connections, the corresponding activation patterns in the mushroom body, say *Y*_1_ and *Y*_2_, happen to be identical, i.e., *Y*_1_ = *Y*_2_. This situation will result in the loss of the information about odor identity, since the two odors cannot be distinguished based on the KC activation pattern alone even if the decoding would be perfect.

In fact, a hypothetical decoder of the odor identity from the MB representation usually should generalize for patterns that are very similar. If a classification is to be made whether *Y*_1_ and *Y*_2_ are representations of different odors or noisy version of the same odor, classifiers would usually make a decision based on the similarity of *Y*_1_ and *Y*_2_. If one defined the similarity by counting the amount of KCs responding differently, i.e., *D*(*Y*_1_, *Y*_2_) ≡ |*Y*_1_ − *Y*_2_|^2^, the classifier might decide that both odors are different if at least *k* KCs response differently, *D*(*Y*_1_, *Y*_2_) ≥ *k*, otherwise the classifier might assume that odors are identical and the minor difference of *Y*_1_ and *Y*_2_ is a result of noise.

How *k* should be chosen depends on the distribution of odors which has to be detected as well as the classifier used for distinguishing odor representations in MB and is thus unknown. However, to enable the readout of the information from the MB representation, the olfactory system should be designed to minimize the probability that AL activations to distinct odors, *X*_1_ ≠ *X*_2_, result in MB representations that are very similar, e.g., *D*(*Y*_1_, *Y*_2_) < *k*.

Such faithful transmission of the odor identity will set constraints on the design of the network, e.g., on the sparsity of the MB activation and on the connectivity structure (〈*C*〉). For example, if the firing threshold θ is small or the number of input connections large, many KCs fire regardless of the pattern in AL. Thus, MB would lose its selectivity to odors: the probability of having two similar KC patterns in response to two distinct odors in AL becomes large. Conversely, if the firing is too sparse, KCs may not respond at all, regardless of the odor. The information about the odor identity would equally be lost.

In the following, we compute the probability that two distinct patterns in AL, *X*_1_ ≠ *X*_2_, result in MB activations, *Y*_1_ and *Y*_2_, that are far apart, *D*(*Y*_1_, *Y*_2_) ≥ *k*. This probability *p*(*D*(*Y*_1_, *Y*_2_) ≥ *k*|*X*_1_ ≠ *X*_2_) is equal to 1 minus the probability that the MB activations are very similar:

(32) $$p\left(D(Y_{1},Y_{2})\geq k|X_{1}\neq X_{2}\right)=1-p(D(Y_{1},Y_{2})<k|X_{1}\neq X_{2})\;\;\;$$

Because exactly *A* glomeruli in the AL are activated in response to an odor, and since each glomerulus contains *M* sister cells (with identical responses), the condition *X*_1_ ≠ *X*_2_ is equivalent to requiring that the number of commonly activated glomeruli is less then *A*. We call these glomeruli the “overlapping” glomeruli between *X*_1_ and *X*_2_ and write *p*(*o*) for the probability of having *o* overlaps. It is

(33) $$p(D(Y_{1},Y_{2})<k|X_{1}\neq X_{2})=\sum_{o=\;0}^{A-\;1}p(D(Y_{1},Y_{2})<k|o)p(o).\;\;$$

The probability *p*(*o*) can be computed as follows. An odor activates *A* glomeruli. There exist $\left(\begin{array}{c} N_{G}\\ A \end{array}\right)$ ways to select those out of the altogether *N*_*G*_ glomeruli. Assume that *o* of the *A* glomeruli are shared between the two odors. Since there are $\left(\begin{array}{c} A\\ o \end{array}\right)\left(\begin{array}{c} N_{G}-A\\ A-o \end{array}\right)$ ways to choose exactly *o* common glomeruli and *A* − *o* non-shared glomeruli, the probability of finding exactly *o* commonly activated glomeruli in both odors is therefore

(34) $$p(o)=\frac{\left(\begin{array}{c} A\\ o \end{array}\right)\left(\begin{array}{c} N_{G}-A\\ A-o \end{array}\right)}{\left(\begin{array}{c} N_{G}\\ A \end{array}\right)}.$$

Because each KC receives uncorrelated synaptic connections from PNs, the probability *p*(*D*(*Y*_1_, *Y*_2_) < *k*|*o*) in Eq. 33 can be further decomposed in terms of individual KCs

(35) $$p(D(Y_{1},Y_{2})<k|o)=\sum_{l<k}p(D(Y_{1},Y_{2})=l|o)$$

(36) $$\begin{array}{l} \;=\sum_{l<k}\left(\begin{array}{c} N_{K}\\ l \end{array}\right){\left(1-p\left(y_{1}=y_{2}|o\right)\right)}^{l}\\ \;p{\left(y_{1}=y_{2}|o\right)}^{N_{K}-l} \end{array}$$

It is thus enough to compute the probability that one KC is commonly activated by two odors, *p*(*y*_1_ = *y*_2_|*o*). This probability is given by adding the probability that two odors both evoke firing, *y*_1_ = *y*_2_ = 1, and the probability that both do not evoke firing, *y*_1_ = *y*_2_ = 0. Hence

(37) $$p(y_{1}=y_{2}|o)=p(y_{1}=1,y_{2}=1|o)+p(y_{1}=0,y_{2}=0|o).\;$$

In the Method Section 4.1, we show how *p*(*y*_1_ = 1, *y*_2_ = 1|*o*) can be calculated. *p*(*y*_1_ = 0, *y*_2_ = 0|*o*) is computed analogously (by changing the range of summation in Eq. 42 accordingly).

Taken together, we have found an expression for the probability that two odor representations differ by at least *k* KC activations in MB, conditioned on whether they are distinct in AL, namely *p*(*D*(*Y*_1_, *Y*_2_) ≥ *k*|*X*_1_ ≠ *X*_2_).

**Figure 6:**
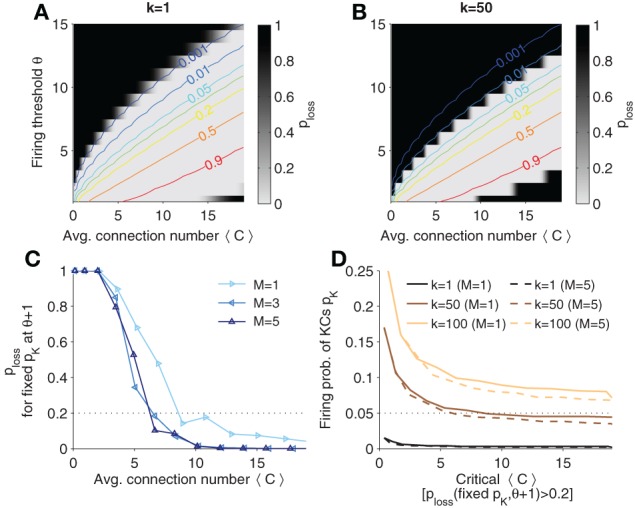
**Probability that distinct odor patterns in AL project to MB patterns less than *k* apart, *p*_loss_ = *p*(*D*(*Y*_1_, *Y*_2_) < *k*|*X*_1_ ≠ *X*_2_). (A,B)** Contour plots of *p*_loss_ for *k* = 1 and *k* = 50, respectively. Colored lines are (smoothed) isolines of constant firing probability *p*_*K*_. **(C)** Probability *p*_loss_ evaluated at iso-lines of constant *p*_*K*_ = 0.05 (as indicated in **B**; here *k* = 50), but with θ increased by 1. Note that the probability increases above 0.2 if 〈*C*〉 is too low (dotted line). **(D)** Sparsest allowable code. Plotted is the critical 〈*C*〉 against levels of firing probability *p*_*K*_ for different *k* and *M*. A firing probability below the indicated lines would result in a high *p*_loss_ (when θ is varied by one). The critical 〈*C*〉 is determined as the point when crossing the threshold (as exemplified in **C**). Colors show different values of *k*. Sister cells do not affect much the maximal allowable sparseness (compares solid to dashed lines). Parameters: *N*_*K*_ = 2000, *N*_*G*_ = 50, *A* = 20.

In Figures 6A, B, the probability of having two similar patterns in MB when AL patterns are different (termed *p*_loss_, “probability of information loss” in the following), *p*(*D*(*Y*_1_, *Y*_2_) < *k*|*X*_1_ ≠ *X*_2_), is plotted in contour plots for different thresholds of *k*, respectively. Lines of constant KC firing probability are plotted in color code. Note that *p*_loss_ is high (dark areas) if the firing threshold is chosen too large (KCs firing is too sparse). Analogously, if firing is too strong, *p*_loss_ is high again.

If one gradually decreases the firing threshold for fixed average synaptic inputs 〈*C*〉, one reaches an area where the probability of information loss quickly vanishes (white areas) and remains near zero. In this area the network can accomplish faithful information processing in a sense that distinct patterns in AL are distinct in MB (from the view point of a readout) with high probability.

The probability *p*(*D*(*Y*_1_, *Y*_2_) ≥ *k*|*X*_1_ ≠ *X*_2_) yields constraints if one has requirements on the discriminability of odors in the MB representation. If the probability is high, distinct odor activation in AL are likely to project to dissimilar representations in MB, so that a classifier could well discriminate odors. The larger *k* the more dissimilar MB representations are required, and the easier would a classifier be able to discriminate between odor patterns. On the other hand, for larger *k* generalization capabilities improve as well, because noisy versions of the odor pattern in MB would generalize to the same odor if less than *k* KCs fire differently. From Figure 6B we see that higher *k* = 50 shifts the probability landscape (as compared to *k* = 1 in Figure 6A) and this sets different constraints on sparseness.

In the previous section, we showed that sparse codes separate patterns. What is the sparsest code allowed? To quantify the sparsest possible firing rate for given *k*, we fixed *p*_*K*_ and looked at *p*_loss_ for corresponding θ and 〈*C*〉. Intuitively, the sparsest code will be on the edge of the white area in Figure 6B. For instance, the line of *p*_*K*_ = 0.05 is very close too the border (see Figure 6B). However, if the code is too sparse, then *p*_loss_ will change dramatically for a small perturbation in e.g., θ. To find the sparsest code, we thus evaluated *p*_loss_ on the line of constant *p*_*K*_ and increase the corresponding θ by one. This probability is plotted for different sister cell numbers in Figure 6C (with *p*_*K*_ = 0.05 and *k* = 50). One notes that the sparsest allowable code depends on the connectivity as well. We define the sparsest allowable code as the one where the probability *p*_loss_ crosses a threshold, *p*_loss_ = 0.2 (see dotted line in Figure 6C). For instance, *p*_*K*_ = 0.05 is the sparsest allowable firing probability when 〈*C*〉 ≈ 5 with little dependence on the sister cell number. In other words, if *p*_*K*_ = 0.05 the average number of connection cannot be below 〈*C*〉 ≈ 5 if the system has to ensure discriminability with *k* = 50.

In Figure 6D is shown how *k* affects the highest allowable sparsity level as well as the minimal connectivity 〈*C*〉 (as estimated as described in Figure 6C). If *k* is increased, denser codes are required. For instance, if 〈*C*〉 = 10 and it is required that at least *k* = 100 KC cells fire differently for distinct odors, the firing probability *p*_*K*_ should be at least 0.1 to ensure that *p*_loss_ is low.

## 3. Discussion

### 3.1. Sister cells increase robustness

We have built a feed-forward network model to understand the organization principles of the drosophila olfactory system. By analyzing the structure of the network and the effect of intrinsic and extrinsic noise, we found that homotypic projection neurons (referred to as sister cells in this paper) are particularly helpful in promoting the robustness of KCs' sparse code to extrinsic noise. Extrinsic noise is here modeled as random activations or inactivation of projection neurons perturbing the mushroom body's odor representation. While increasing the sheer number of PNs used for the odor representation in AL increases the robustness to noise as well, it turns out that inserting sister cells is much more efficient as the noise vanishes with 1/*M* (*M* being the number sister cells per glomerulus). The crucial mechanism is the strong gap-junction coupling of sister cells, which increases the correlation of sister cells and therefore reduces the probability of accidental activations or inactivation.

Although the strength of noise the system has to cope with, as well as the level of noise which is still tolerable in the MB representation, is unknown, our model suggests that a few sister cells per glomerulus (e.g., 4–5) might be sufficient given the strong 1/*M* noise dependence. This result is consistent with the experimental literature, where typically 2–5 highly correlated sister cells are found per glomeruli in drosophila (Stocker et al., 1990).

Other species might use different strategies than relying on sister cells to tackle noise. For instance, in locusts the number of PNs is large [≈830 (Leitch and Laurent, 1996)] and they seem to lack sister cells similar to those of flies (Martin et al., 2011). As mentioned, increasing the sheer amount of PNs indeed increases robustness to noise, although to a lesser degree. Therefore, additional mechanism might be necessary to reduce noise in the locust. Indeed, it has been reported that a wild field interneuron in the locust MB can improve the robustness of sparse code via a feedback modulation (Papadopoulou et al., 2011) suggesting that the locust olfactory system might have a different strategy.

Although evidences for sister cells in other insects remain few, there are, however, species where cells anatomically similar to sister cells in drosophila have been described. For instance, in frog similar gap-junction coupled cells in glomeruli have been found (coherent mitral/tufted cells) (Chen et al., 2009). Interestingly, each glomeruli only had a few cells (2–7) comparable to the situation in flies suggesting that these cells might have a similar functional role in the reduction of extrinsic noise. Another example are mitral cells in mice, which are also anatomically comparable to sister cells in drosophila as they receive inputs from the same glomeruli and project to an analogous structure to the KCs (Dhawale et al., 2010; Padmanabhan and Urban, 2010; Tan et al., 2010). However, in contrast to drosophila, where sister cells receive almost identical feed-forward input from ORNs and are additionally electrically coupled, mitral cells in mice have an intrinsic biophysical diversity (Padmanabhan and Urban, 2010) and are embedded into an inhibitory network causing more complex temporal dynamics (Dhawale et al., 2010; Tan et al., 2010). Thus, since mitral cells seem not to be correlated as strongly as drosophila sister cells, their functional role in the olfactory system of mice is likely to be different and not directly comparable to sister cells in drosophila.

Intrinsic noise variability, on the other hand, is less dependent on the number of sister cells. Although variability reduces when introducing sister cells (mainly because of a slight broadening of the synaptic input distribution) the effect saturates quickly with increasing sister cell number. We thus conclude that other strategies than increasing sister cell number might be employed to cope with intrinsic noise.

Interestingly, the average number of connections a KC receives, 〈*C*〉, has opposite influence on intrinsic or extrinsic noise. In principle, a trade-off of importance of both types of noises would thus allow to find an optimal value for the connectivity number. However, the strengths and importance weightings of the respective noise types, as well as potential other biological constraints on the connectivity structure, are unknown, so that calculating the “most robust” connectivity number remains elusive. However, different species potentially are exposed to different degrees of noises and thus might optimize the connectivity number according to their requirements. For instance, in contrast to the fly, it was found that in locust KCs receive inputs from almost 50% of the PNs (Jortner et al., 2007). This high connectivity would favor the reduction of intrinsic over extrinsic noise. One might speculate that the locust with its greater amount of neurons in the olfactory system (Leitch and Laurent, 1996) is more challenged by intrinsic noise rather than extrinsic noise and thus might have evolved a high connectivity.

### 3.2. Computational aspects

A well accepted concept from machine learning and reservoir computing is that a divergent projection enables a system to improve its computation capabilities as representations are re-coded into a higher dimensional space, where representations become well separated, so that the extraction of information often requires less sophisticated read-outs, e.g., linear instead of non-linear classifiers (Maass et al., 2002; Bishop and Nasrabadi, 2006; Jaeger, 2007; Barak et al., 2013). Accordingly, in our model of the olfactory system, the divergent projection helps to separate AL representations when projected into the MB: we show that for sparse KC firing the number of shared activations in two odors are reduced (see Figure 5).

Taking the connectivity constraints of the drosophila olfactory system into account, such as a random, sparse, and purely excitatory projection, we further derived the probability of whether distinct input patterns (in AL) have similar representations in MB (the “reservoir”). This probability is at the core of a hypothetical read-out trying to discriminate between two odors: the parameter *k* regulates how far separated distinct representations in MB have to be. Any classifier of the neural responses will in some sense rely on the distance to distinguish responses to two odor patterns. Thus, higher *k* potentially improves the discrimination capabilities. We found that requiring a large separation of odor patterns puts constraints on the sparseness of the representation. Too sparse codes will not provide the required distance. For instance, if fewer than *k*/2 neurons are activated to two odors, the number of differently activated neurons is naturally lower than *k*, so that two odor patterns become too close. Furthermore, the lowest allowable firing probability also constrains the connectivity: having too few input connections will not allow the system to achieve a required firing level for a stable odor representation.

If one assumes very low level of firing probability *p*_*K*_ as suggested by recent evidence (Campbell et al., 2013), for instance *p*_*K*_ = 0.05, one can attempt to make a prediction on the minimal number of connections 〈*C*〉. Although this critical number of connections depends on the discrimination requirements (parameter *k*), we find that e.g., for *k* = 50 (that is 2.5% of the KCs have to be different for distinct odors) the average number of connections cannot be chosen smaller than 〈*C*〉 ≈ 6 − 8 (depending somewhat on the number of sister cells; see Figure 6C). This value agrees with experimental literature, where 10 inputs per KC (i.e., each KC receives connections from about 6.6% of the PNs) was measured (Turner et al., 2008) suggesting that the olfactory system might operate close to the maximally allowed sparsity level.

It is well known that discrimination ability trades-off with generalization capability (Barak et al., 2013). In this sense, larger *k* allow also for better generalization, because nearby patterns in MB could be assigned as noisy version of the same odor. Apart from this viewpoint of a classifier extracting the information from the output network alone, other strategies for better generalization capabilities might be implemented in neural systems. Here we put forward a new hypothesis how better generalization capabilities might be achieved in the specific case of drosophila: by the insertion of highly correlated sister cells into glomeruli to enhance noise robustness already at the level of the inputs.

### 3.3. Assumption of the model framework

We analyzed the design principles of the AL to MB projection in the drosophila. Our mathematical model is similar to the mathematical framework developed previously for analyzing the olfactory network in case of locusts (García-Sanchez and Huerta, 2003). While building on the earlier work, some crucial aspects of the drosophila network are different to the locust. In particular, lateral inhibition between glomeruli ensures that the total activity of PNs to any odor is roughly constant in flies (Luo et al., 2010; Olsen et al., 2010). Regarding thus the number of activated glomeruli as fixed (the parameter *A*) allowed us to derive analytical expressions and approximations, in contrast to the mainly numerical evaluation of the earlier study in locust (García-Sanchez and Huerta, 2003). Moreover, we here develop methods for investigating robustness to noise and focus on the special role of sister cells in the fly which are absent in locust.

Our network model makes several assumptions. First, we assumed that the sparse coding in MB is mainly determined by feedforward synaptic input and not for instance by recurrent processing in MB. Experimental evidences suggest that this assumption is biologically plausible, because the influence of (potential) recurrent interaction seem to be weak (Turner et al., 2008). Recently, another study reporting that the KC firing activity can be described by a linear combination of PN inputs together with a threshold mechanism points in the same direction (Li et al., 2013).

Second, sister cells in our model project independently to KCs in MB, which seem to be well supported by experimental data (Masuda-Nakagawa et al., 2005; Kazama and Wilson, 2009; Caron et al., 2013).

Third, the neuron model consists of simple binary neurons and thus neglects any graded activity (firing rate) or temporal structure of the odor representation. In simulations, we found that implementing graded activity in projection neurons does not have influence on the qualitative results. Moreover, experimental evidence suggests that temporal dynamics seems not to play a prominent role in the olfactory coding of flies in contrast to e.g., the locust, where spatio-temporal dynamics might be an important aspect of the code (Wehr and Laurent, 1996; Mazor and Laurent, 2005). Thus, at least for drosophila our approach of coincident spatial coding seems reasonable.

Fourth, we used fixed number of glomeruli to model AL's activity pattern to odorant input. This is reasonable because of the effect of a divisive normalization operation in AL which effectively equalizes the total activity of PNs, regardless of odorant identity and concentration. In reality, the number of activated glomeruli may vary somewhat in response to odorant input. However, this is not likely to affect the main results of our study.

Finally, we did not consider possible variation of the number of sister cells for some selected glomeruli as suggested experimentally (Stocker et al., 1990). However, a variation in the number of sister cells per glomeruli would not change our qualitative results. The prediction of the model would be that variation of sister cells per glomeruli would indicate a disproportional sensitivity to noise for those glomeruli having more sister cells.

## 4. Methods

### 4.1. Derivation of the KC firing probability for given overlap *o*

Here we derive the probability *p*(*y*_1_ = 1, *y*_2_ = 1|*o*) that a KC fires in response to each of two odors which share exactly *o* glomeruli activations in AL.

To respond similarly to both odors a KC has to receive enough synaptic inputs to reach the threshold θ for both odors. Since the input connections are random, one can decompose

(38) $$p(y_{1}=1,y_{2}=1|o)=\sum_{C}p(C)p(y_{1}=1,y_{2}=1|o,C),$$

where the connection structure *p*(*C*) is given by the binomial distribution Eq. 1.

We can decompose further when considering that a KC receives exactly *w*_*c*_ synaptic inputs from the *Mo* commonly activated PNs of *X*_1_ and *X*_2_:

(39) $$p(y_{1}=1,y_{2}=1|o,C)=\sum_{w_{c}}p(y_{1}=1,y_{2}=1|o,w_{c},C)p(w_{c}|o,C)$$

The probability *p*(*w*_*c*_|*o*, *C*) can be calculated as follows. Assume that the KC receives *C* connections from random PNs. There exist $\left(\begin{array}{c} MN_{G}\\ C \end{array}\right)$ ways to connect to *C* of altogether *MN*_*G*_ PNs. Since there are $\left(\begin{array}{c} Mo\\ w_{c} \end{array}\right)\left(\begin{array}{c} MN_{G}-Mo\\ C-w_{c} \end{array}\right)$ ways that exactly *w*_*c*_ of the *C* connection come from the overlapping glomeruli and the other *C* − *w*_*c*_ connections come from the *MN*_*G*_ rest PNs, one can write

(40) $$p(w_{c}|o,C)=\frac{\left(\begin{array}{c} Mo\\ w_{c} \end{array}\right)\left(\begin{array}{c} MN_{G}-Mo\\ C-w_{c} \end{array}\right)}{\left(\begin{array}{c} MN_{G}\\ C \end{array}\right)}.$$

Note that *p*(*w*_*c*_|*o*, *C*) follows a hypergeometric distribution, which describes the probability of *w*_*c*_ successes in *C* draws without replacement from population size *MN*_*G*_ containing *Mo* wins.

**Figure 7:**
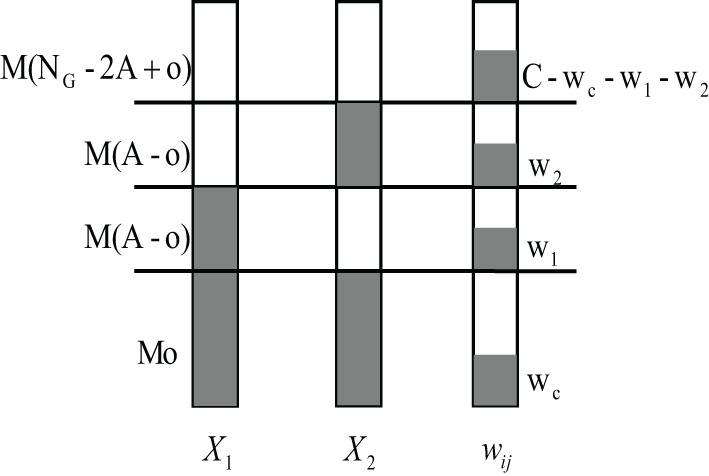
**Schematic diagram of calculating the joint probability *p*(*w*_1_, *w*_2_|*o*, *w*_*c*_, *C*).** Suppose two input pattern *X*_1_ and *X*_2_ have *o* overlapping activated glomeruli, and the *i*th KC connects to *w*_*c*_ PNs within these *Mo* shared PNs. The KC connects altogether to *C* PNs. It thus additionally receives *w*_1_ and *w*_2_ inputs from the rest of the *M*(*A* − *o*) non-overlapping but activated PNs of pattern *X*_1_ and *X*_2_, respectively. Finally, *C* − *w*_*c*_ − *w*_1_ − *w*_2_ connections come from non-activated PNs.

We now look closer at the probability *p*(*y*_1_ = 1, *y*_2_ = 1|*o*, *w*_*c*_, *C*) in Eq. 39. Apart from the *w*_*c*_ active synaptic inputs from the shared glomeruli, the KC still gets other inputs from active glomeruli that are not shared. Assume that it receives *w*_*1*_ of the altogether *C* inputs from the *M*(*A* − *o*) other activated glomeruli of odor 1 and *w*_2_ inputs from the *M*(*A* − *o*) other activated glomeruli of odor 2, and the rest *C* − *w*_*c*_ − *w*_1_ − *w*_2_ input connections from the non-activated PNs, we can compute the probability *p*(*w*_1_, *w*_2_|*o*, *w*_*c*_, *C*) as (compare to Figure 7):

(41) $$p(w_{1},w_{2}|o,w_{c},C)=\frac{\left(\begin{array}{c} MA-Mo\\ w_{1} \end{array}\right)\left(\begin{array}{c} MA-Mo\\ w_{2} \end{array}\right)\left(\begin{array}{c} MN_{G}-2MA+Mo\\ C-w_{c}-w_{1}-w_{2} \end{array}\right)}{\left(\begin{array}{c} MN_{G}-Mo\\ C-w_{c} \end{array}\right)}.$$

Finally, *p*(*y*_1_ = 1, *y*_2_ = 1|*o*, *w*_*c*_, *C*) can be computed by adding the probability of all cases where the amount of input reaches the threshold, namely *w*_*c*_ + *w*_1_ ≥ θ and *w*_*c*_ + *w*_2_ ≥ θ:

(42) $$p(y_{1}=1,y_{2}=1|o,w_{c},C)=\sum_{w_{2}\geq \theta -w_{c}}\sum_{w_{1}\geq \theta -w_{c}}p(w_{1},w_{2}|o,w_{c},C).$$

Taken together we have derived the probability *p*(*y*_1_ = 1, *y*_2_ = 1|*o*).

### 4.2. Response variability to general extrinsic noise

In the main text we did only consider that *m* previously quiescent PNs are activated as a result of noise. However, in principle, previously activated cells might also become silent because of the noise perturbation. Here we derive this more general case of extrinsic noise.

Assume that *m*_1_ active PNs become silent and *m*_2_ quiescent PNs activate in response to a noise perturbation. We calculate the change in the firing probability KC. Suppose a KC receives *n* inputs without noise. We suppose that as a result of the noise perturbation, *m*_1_ previously activated neurons become silent. The probability that inputs get reduced by *n*_1_ when silencing *m*_1_ PNs is given by the chance that the *m*_1_ cells are one of those *n* which are connected to the KC (hypergeometric distribution)

(43) $$p(n_{1}|n)=\frac{\left(\begin{array}{c} m_{1}\\ n_{1} \end{array}\right)\left(\begin{array}{c} MA-m_{1}\\ n-n_{1} \end{array}\right)}{\left(\begin{array}{c} MA\\ n \end{array}\right)}.$$

If *m*_2_ quiescent neurons become activated, the probability of a KC receiving *n*_2_ additional synaptic inputs from these additionally activated PNs follows the binomial distribution

(44) $$p(n_{2})=\left(\begin{array}{c} m_{2}\\ n_{2} \end{array}\right)p_{c}^{n_{2}}{\left(1-p_{c}\right)}^{m_{2}-n_{2}}.$$

Finally, to get the total noise induced change in firing probability Δ*p*^extr^_*K*_, we have to add the probabilities that it was *n* < θ before but it is *n* − *n*_1_ + *n*_2_ ≥ θ after perturbation (more firing), as well as the cases when it was *n* ≥ θ before and *n* − *n*_1_ + *n*_2_ < θ after perturbation (less firing):

(45) $$\begin{array}{l} \Delta p_{{}_{K}}^{\text{extr}}=\sum_{n<\theta }p(n)p\left(n-n_{1}+n_{2}\geq \theta \right)\\ \;+\sum_{n\geq \theta }p(n)p\left(n-n_{1}+n_{2}<\theta \right) \end{array}$$

(46) $$\begin{array}{l} \;=\sum_{n\;<\theta }p(n)\sum_{n-n_{1}+n_{2}\geq \theta }p\left(n_{1}|n\right)p\left(n_{2}\right)\\ \;+\sum_{n\geq \theta }p(n)\sum_{n-n_{1}+n_{2}<\theta }p\left(n_{1}|n\right)p\left(n_{2}\right) \end{array}$$

Note that in our case noise induced silencing or activating cells is independent, i.e., *p*(*n*_1_, *n*_2_|*n*) = *p*(*n*_1_|*n*)*p*(*n*_2_).

### 4.3. Simulation of graded responses in AL

As we have only considered binary neurons in the main text for mathematical simplicity, we here analyze in a simulation whether results can be generalized to neurons with graded activities.

In fact, to use binary neurons is a simplification as PNs respond with a certain firing rate when presented with an odor. In this section, we tested whether graded activities would change the main results on noise robustness as derived for binary cells in the main text.

Each odor is represented with a subset of activated glomerulus *A*. We assume that non-activated PNs are silent, i.e., their firing rate is 0. Considering that the total sum of rates of all activated PNs in response to an odor stimulation is approximately constant [because of lateral divisive normalization (Luo et al., 2010)], we set the firing rate *x*_*i*_ of an activated PN of the *i*th glomerulus to

(47) $$x_{i}=R\frac{\xi_{i}}{\sum_{j}\xi_{j}},$$

where ξ_*i*_ is a random number obeying a binomial distribution (with parameters *N* and *p*), and *R* is a parameter determining the response range of the PNs. The normalization by the sum of all ξ_*i*_ ensures that the total activity to an odor is constant and equals to *MR*. Note that all sister cells have the same activation level. The connectivity structure from AL to MB is identical to that described in the main text.

As a result of noise, response patterns potentially change. To quantitative this change, we define the response dissimilarity

(48) $$E=\frac{|Y^{\prime }-Y{|}^{2}}{2|Y{|}^{2}}$$

of a KC pattern *Y* ≠ 0 with its noise perturbed version *Y*′. Note that *E* is zero if *Y*′ = *Y* and is smaller than 1 if *Y*′ differs by less than the number of activated KCs in *Y*. In the numerical simulations, we randomly apply 1000 input patterns and calculate the response of KCs in case of noise and without. Then we calculate the sample mean and standard error of the dissimilarities *E*. When varying parameters (such as *M* and 〈*C*〉), we hold the firing probability of KCs fixed to 0.05 as done in the case of binary neurons.

#### 4.3.1. Intrinsic noise

**Figure 8:**
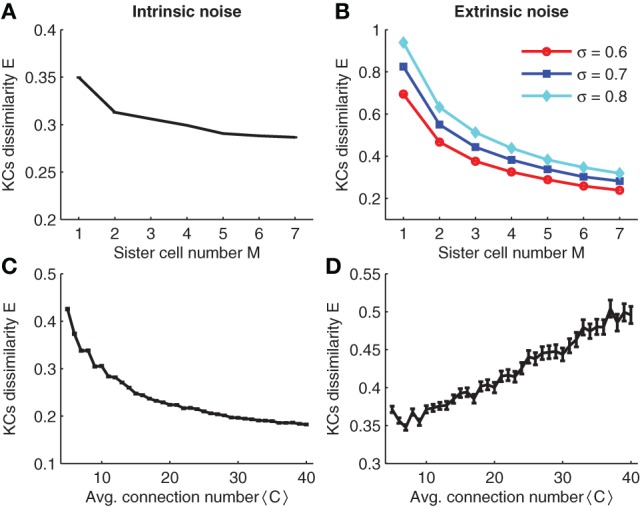
**(A,C)** Effect of intrinsic noise on KCs dissimilarity with respect to sister cell number and average input connection number. **(B,D)** Effect of extrinsic noise on KCs dissimilarity with respect to sister cell number and average input connection number. Parameters: *N*_*G*_ = 50,*A* = 20,*N* = 100, *p* = 0.3, *p*_*K*_ ≈ 0.05, σ_intr_ = 40,σ_extr_ = 0.6 in **(D)**,<*C*> = 10 in (**A** and **C**), and *M* = 3 in **(B,D)**.

In the simulations, intrinsic noise is a perturbation of the KC firing threshold, i.e., θ → θ + Δθ where Δθ is a Gaussian random number with zero mean and standard deviation σ_intr_. The result of the simulation is plotted in Figure 8A. Note that for different sister cell numbers *M*, the variability decays in a similar manner as for the binary case (compare Figures 8A and 3A).

#### 4.3.2. Extrinsic noise

We next tested the effect of extrinsic noise on KC firing variability. Extrinsic noise is introduced by adding Gaussian noise fluctuations to the activity of PNs. That is, the activity in the *i*th glomeruli in response to odor stimuli is changed to $x_{i}\to x_{i}+\sigma_{\text{extr}}/\sqrt{M}x_{i}\eta_{i}$, where η_*i*_ is a Gaussian random number (zero mean and unit variance). Note that the standard deviation of the extrinsic noise term is linearly dependent on the mean firing activity ξ_*i*_ to ensure a constant coefficient of variation. Furthermore, the term $\sqrt{M}$ ensures that the variation of the noise decreases in the correct manner if strong gap-junction couplings are considered (see Section 2.5).

Simulation results are shown in Figure 8B. Note that sister cells promote the robustness to extrinsic noise in a similar manner as shown for binary neurons (compare to Figure 3B). Analogous to the case of binary neurons, increasing the average number of connections 〈*C*〉 has opposite effects on intrinsic and extrinsic noise: larger 〈*C*〉 reduces intrinsic noise while smaller 〈*C*〉 enhances robustness to extrinsic noise (compare Figures 8C, D).

### Conflict of interest statement

The authors declare that the research was conducted in the absence of any commercial or financial relationships that could be construed as a potential conflict of interest.
